# Supplementary material for: The protease corin regulates electrolyte homeostasis in eccrine sweat glands
Source: PLoS Biol. 2021 Feb 16;19(2):e3001090. doi: 10.1371/journal.pbio.3001090 (PMC7909636; doi:10.1371/journal.pbio.3001090)
Supplement: S1 Fig — (A) Immunohistochemical analysis of corin expression in human scalp sections. Positive corin staining (brown) was detected in hair follicles. A boxed area is shown below in a higher magnification. Scale bars are indicated. (B) HE, corin, and cytokeratin staining in serial human scalp sections. A normal IgG was used as a negative control in immunohistochemical analysis. Boxed areas are shown below in a higher magnification. Scale bars are indicated. Data are representative of at least 3 experiments. HE, hematoxylin–eosin; IgG, immunoglobulin G. (PDF) [file pbio.3001090.s001.pdf]

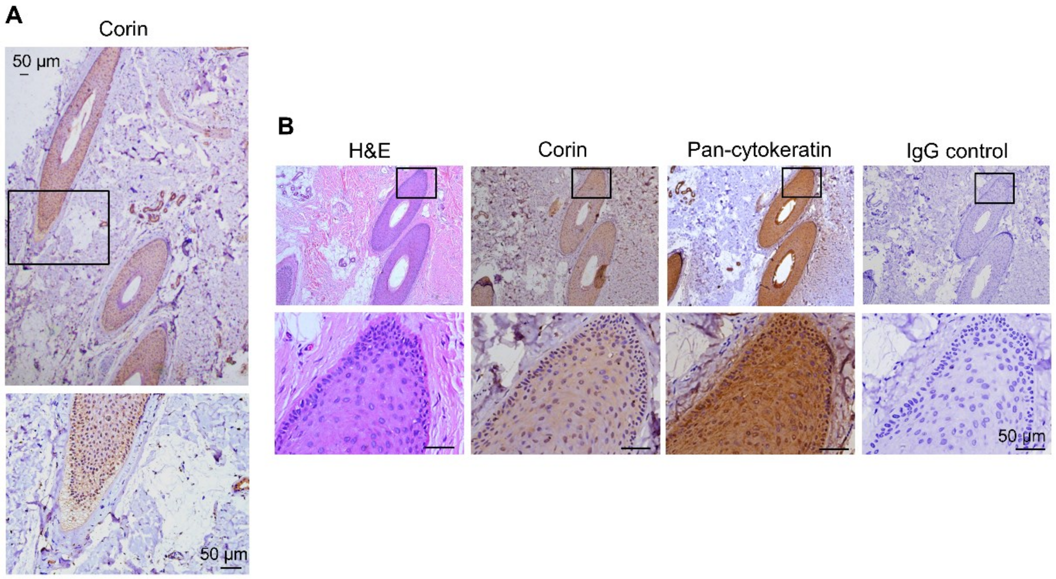

S1 Fig. Corin expression in human hair follicles. (A) Immunohistochemical analysis of corin expression in human scalp sections. Positive corin staining (brown) was detected in hair follicles. A boxed area is shown below in a higher magnification. Scale bars are indicated. (B) H&E, corin and cytokeratin staining in serial human scalp sections. A normal IgG was used as a negative control in immunohistochemical analysis. Boxed areas are shown below in a higher magnification. Scale bars are indicated. Data are representative of at least three experiments.
