# Supplementary material for: The protease corin regulates electrolyte homeostasis in eccrine sweat glands
Source: PLoS Biol. 2021 Feb 16;19(2):e3001090. doi: 10.1371/journal.pbio.3001090 (PMC7909636; doi:10.1371/journal.pbio.3001090)
Supplement: S2 Fig — (A) Illustration of the strategy to disrupt the Corin gene by inserting 2 loxP sites flanking exon 4. PCR primers used in genotyping are indicated. (B–D) PCR analysis using indicated oligonucleotide primers to identify mice with Corflox and Cordel4 alleles before (B and C) and after (D) exon 4 was deleted by crossing with mice expressing Cre. KO, knockout. (PDF) [file pbio.3001090.s002.pdf]

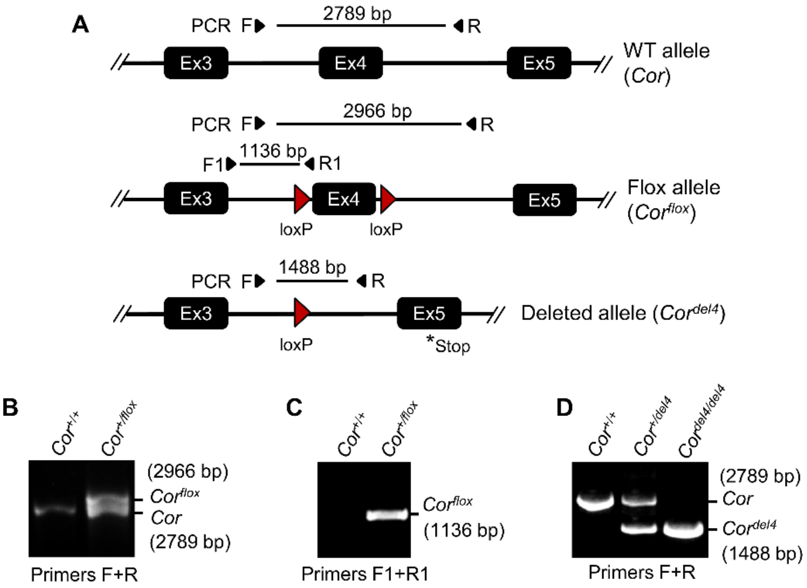

S2 Fig. Generation of corin KO mice. (A) Illustration of the strategy to disrupt the *Corin* gene by inserting two loxP sites flanking exon 4. PCR primers used in genotyping are indicated. (B-D) PCR analysis using indicated oligonucleotide primers to identify mice with *Cor*^{flox} and *Cor*^{del4} alleles before (B and C) and after (D) exon 4 was deleted by crossing with mice expressing *Cre*.
