# Supplementary material for: The protease corin regulates electrolyte homeostasis in eccrine sweat glands
Source: PLoS Biol. 2021 Feb 16;19(2):e3001090. doi: 10.1371/journal.pbio.3001090 (PMC7909636; doi:10.1371/journal.pbio.3001090)
Supplement: S3 Fig — (A and B) Immunohistochemical staining of corin (A), ANP, and NPR-A (B) in footpad sections from WT and corin KO mice. In (A), eccrine sweat glands are indicated by red arrowheads. In (B), positive ANP and NPR-A staining in epithelial cells are indicated by black arrowheads. Normal IgG was used as a negative control. Scale bars are indicated. (C) Levels of Npr1 mRNA levels in footpads from WT and corin KO mice, analyzed by quantitative RT-PCR. n = 8 mice per group. (D) Footpad sections from WT and corin KO mice were stained with HE. Eccrine sweat glands are indicated by red arrowheads. No changes in eccrine sweat gland structure and numbers were observed between WT and corin KO mice. Data are representative of at least 3 experiments. (E) Illustration of the iodine–starch test used in this study. Mouse paws were cleaned and coated with iodine and starch. Pilocarpine, a sweat stimulant, was injected, s.c., in paws. Photos were taken at 0 (control), 1 (for sweat gland numbers), and 2 min (for sweat excretion) after the injection. (F) Representative photos taken at 1 min after pilocarpine injection in WT and corin KO mice. (G) Quantitative data of black-staining areas in WT and corin KO mouse paws are presented as mean ± SEM. n = 10 mice per group. Data in C and G were analyzed with 2-tailed Student t test and Mann–Whitney test, respectively, and the original numerical values are in S1 Data. ANP, atrial natriuretic peptide; HE, hematoxylin–eosin; IgG, immunoglobulin G; KO, knockout; NPR-A, natriuretic peptide receptor-A; RT-PCR, reverse transcription PCR; WT, wild-type. (PDF) [file pbio.3001090.s003.pdf]

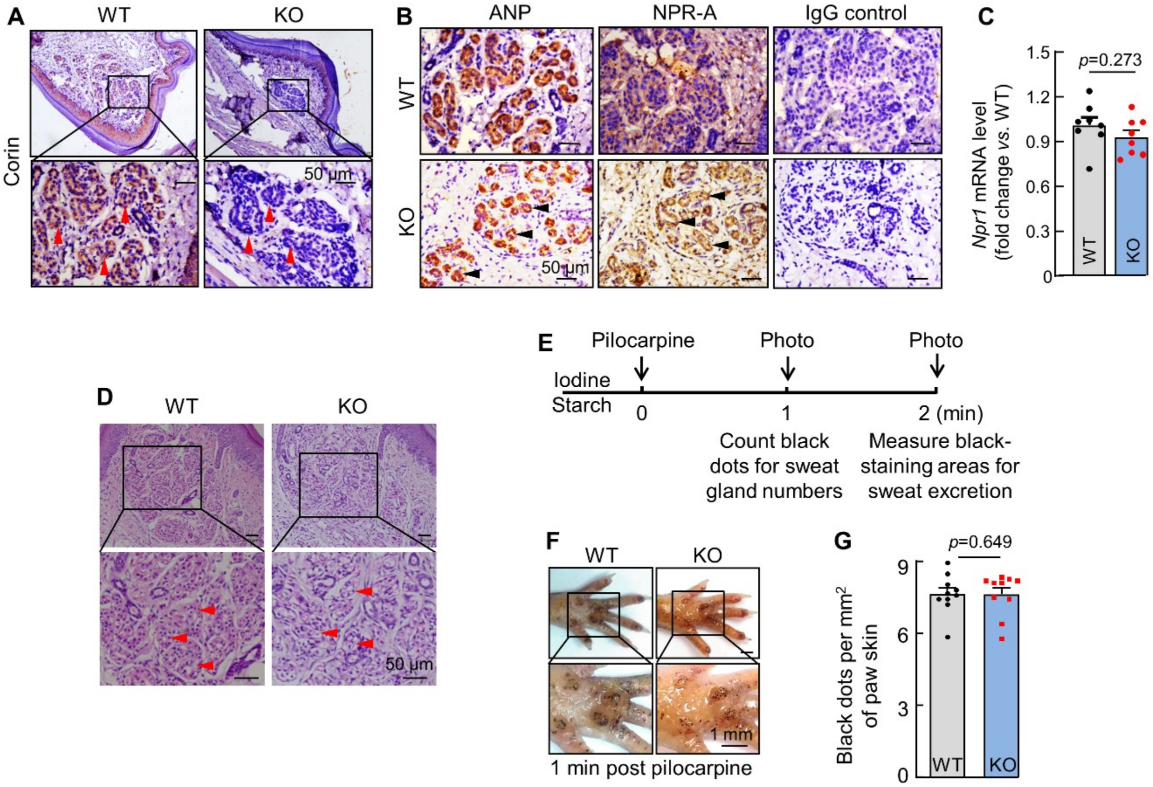

S3 Fig. Corin, ANP and NPR-A expression and analysis of eccrine sweat glands in mouse footpads. (A and B) Immunohistochemical staining of corin (A), ANP and NPR-A (B) in footpad sections from WT and corin KO mice. In (A), eccrine sweat glands are indicated by red arrowheads. In (B), positive ANP and NPR-A staining in epithelial cells are indicated by black arrowheads. Normal IgG was used as a negative control. Scale bars are indicated. (C) Levels of *Npr1* mRNA levels in footpads from WT and corin KO mice, analyzed by quantitative RT-PCR. n = 8 mice per group. (D) Footpad sections from WT and corin KO mice were stained with H&E. Eccrine sweat glands are indicated by red arrowheads. No changes in eccrine sweat gland structure and numbers were observed between WT and corin KO mice. Data are representative of at least three experiments. (E) Illustration of the iodine-starch test used in this study. Mouse paws were cleaned and coated with iodine and starch. Pilocarpine, a sweat stimulant, was injected, *s.c.*, in paws. Photos were taken at 0 (control), 1 (for sweat gland numbers) and 2 min (for sweat excretion) after the injection. (F) Representative photos taken at 1 min after pilocarpine injection in WT and corin KO mice. (G) Quantitative data of black-staining areas in WT and corin KO mouse paws are presented as mean ± SEM. n = 10 mice per group. Data in C and G were analyzed with two-tailed Student's *t* test and Mann-Whitney test, respectively, and the original numerical values are in S1 Data.
