# Supplementary material for: The protease corin regulates electrolyte homeostasis in eccrine sweat glands
Source: PLoS Biol. 2021 Feb 16;19(2):e3001090. doi: 10.1371/journal.pbio.3001090 (PMC7909636; doi:10.1371/journal.pbio.3001090)
Supplement: S4 Fig — (A) Quantitative RT-PCR analysis of Scnn1b mRNA, encoding β-ENaC, in footpads from WT and corin KO mice on 0.3% salt diet. (B) Western blotting of β-ENaC protein in footpads from WT and corin KO mice (n = 4 per group) on 0.3 salt diet. Proteins expression levels were quantified by densitometric analysis of the western blot. (C) Systolic BP in WT and corin KO mice on 0.3% or 4% salt diet treated with vehicle or amiloride. (D) Systolic BP in WT and corin KO mice on 0.3% salt diet treated with vehicle or amlodipine. (E) Sweat excretion was analyzed by the iodine–starch test in WT and corin KO mice on 0.3% salt diet and treated with (+) or without (−) amlodipine and pilocarpine (pilocar). (F) Quantitative RT-PCR analysis of Cftr mRNA, encoding CFTR, in footpads from WT and corin KO mice on 0.3% salt diet. All data are mean ± SEM. P values were analyzed by 2-tailed Student t test (in A, C, and E) or 1-way ANOVA (in B and D). In bar graphs, each dot represents data from 1 mouse, and the original numerical values are in S1 Data. BP, blood pressure; CFTR, cystic fibrosis transmembrane conductance regulator; ENaC, epithelial sodium channel; KO, knockout; RT-PCR, reverse transcription PCR; WT, wild-type. (PDF) [file pbio.3001090.s004.pdf]

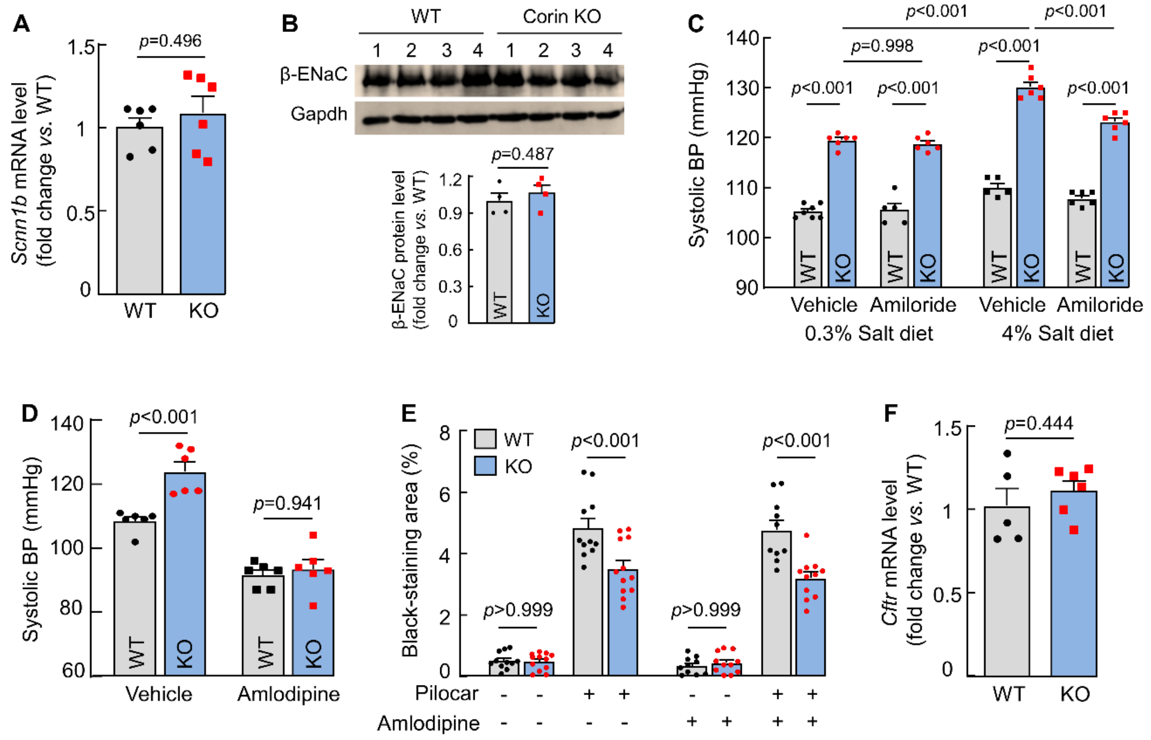

S4 Fig. ENaC and CFTR expression and effects of amiloride and amlodipine on blood pressure and sweat excretion. (A) Quantitative RT-PCR analysis of *Scnn1b* mRNA, encoding β-ENaC, in footpads from WT and corin KO mice on 0.3% salt diet. (B) Western blotting of β-ENaC protein in footpads from WT and corin KO mice (n = 4 per group) on 0.3 salt diet. Proteins expression levels were quantified by densitometric analysis of the western blot. (C) Systolic blood pressure (BP) in WT and corin KO mice on 0.3% or 4% salt diet treated with vehicle or amiloride. (D) Systolic BP in WT and corin KO mice on 0.3% salt diet treated with vehicle or amlodipine. (E) Sweat excretion was analyzed by the iodine-starch test in WT and corin KO mice on 0.3% salt diet and treated with (+) or without (-) amlodipine and pilocarpine (pilocar). (F) Quantitative RT-PCR analysis of *Cftr* mRNA, encoding CFTR, in footpads from WT and corin KO mice on 0.3% salt diet. All data are mean ± SEM. *P* values were analyzed by two-tailed Student's *t* test (in A, C and E), or one-way ANOVA (in B and D). In bargraphs, each dot represents data from one mouse and the original numerical values are in S1 Data.
