# Supplementary material for: The protease corin regulates electrolyte homeostasis in eccrine sweat glands
Source: PLoS Biol. 2021 Feb 16;19(2):e3001090. doi: 10.1371/journal.pbio.3001090 (PMC7909636; doi:10.1371/journal.pbio.3001090)
Supplement: S1 Text — Detailed experimental procedures are described in the Supporting information Materials and methods. Primers used in the study are listed in Table A. Antibodies used in the study are listed in Table B. (DOCX) [file pbio.3001090.s005.docx]

**The Protease Corin Regulates Electrolyte Homeostasis in Eccrine Sweat Glands**

**Supporting Information**

**Supplementary Materials and Methods**

**Corin KO and heart conditional KO mice**

Corin KO mice were generated at the Nanjing Biomedical Institute of Nanjing University (Nanjing, China). A Cas9-based strategy was designed to insert two loxP sites flanking mouse *Corin* gene exon 4 (S2A Fig.). A donor plasmid was made and microinjected into fertilized oocytes to generate mice with a flox *Corin* allele (*Cor^flox^*), which was screened by PCR analysis (S2B and C, Fig.). Sequences of the oligonucleotide primers used in genotyping are listed in S1 Table. The *Cor^+/flox^* mice were selected and bred with the CMV-Cre mice (B6.C-Tg(CMV-cre)1Cgn/J) with a ubiquitously expressed *Cre* gene [1] to generate mice with the null *Corin* allele (*Cor^del4^*) (S2D Fig.), which were referred to as corin KO mice in this study. In RT-PCR and western blotting, corin mRNA and protein were not detected in corin KO mice.

To generate corin heart conditional KO (hcKO) mice, *Cor^+/flox^* mice were crossed with B6.FVB(129)-A1cf^Tg(Myh6-cre/Esr1*)1Jmk^/J mice expressing *Cre* in cardiomyocytes under the myosin heavy chain promoter [2] to disrupt the *Corin* gene specifically in the heart. The offspring was screened by PCR. Corin hcKO mice were verified by RT-PCR analysis. Like corin global KO mice, corin hcKO mice were hypertensive (systolic blood pressure: 117.8 ± 1.5 mmHg *vs*. 107.0 ± 1.7 mmHg in WT mice, *p*<0.001, n = 8 per group; or 117.4 ± 2.9 mmHg in corin global KO mice, *p*=0.934, n = 8 per group). There were no significant differences in plasma Na^+^ and Cl^-^ levels among WT (Na^+^: 145.2 ± 5.4 mM; Cl^-^: 105.1 ± 3.7 mM), corin KO (Na^+^: 149.3 ± 2.7 mM; Cl^-^: 109.0 ± 1.8 mM), and corin hcKO (Na^+^: 150.7 ± 3.8 mM; Cl^-^: 110.0 ± 2.3 mM) mice (n = 4-10 per group; all *p* values >0.05).

All mice were housed in ventilated cages with nesting materials and plastic tunnels and free access to water and food with normal-salt (0.3% NaCl), or high-salt (4% NaCl). The specific-pathogen-free animal facility was temperature-controlled with 12:12-hour light-dark cycles. WT and KO mice used were not littermates but were bred and housed in the same room at the facility. Age- and gender-matched mice (equal numbers of males and females; 8-12 weeks old, 20-30 grams), unless indicated otherwise, were randomized into test groups. The sample size per study group was based on published comparable studies, pilot experiments, and statistical power analysis. The investigators who performed the experiments were not blinded to mouse genotypes.

Immune staining

For immunohistochemical analysis, skin tissues were fixed with 4% (v/v) paraformaldehyde and embedded in paraffin. Serial sections in 4-μm thickness were cut, mounted on adhesion microscope slides (Citotest Scientific), and used alternately for staining with antibodies against different proteins. The sections were de-paraffinzed with xylene (15 min, room temperature), re-hydrolyzed with graded ethanol solutions (3 min each), and boiled in a sodium citrate solution (10 mM, pH 6.0) for antigen retrieval. Hydrogen peroxide (3% in a methanol solution) was used to inhibit endogenous peroxidase activity [3]. After incubation with 5% bovine serum albumin (BSA) in phosphate-buffered saline (PBS) (30 min, 37°C) to block non-specific binding, sections were incubated with primary antibodies at 4°C overnight. Primary antibodies used in this study included those against corin (made in the lab, 1:500 dilution) [4], pro-ANP/ANP (Abcam, ab85304, 1:100 dilution), NPR-A (Abcam, ab14356, 1:1000 dilution), β-ENaC (StressMarq Biosciences, spc-404, 1:100 dilution), CFTR (Abcam, ab2784, 1:200 dilution), pan-cytokeratin (MXB Biotechnologies, kit-0009, as provided), SMA (Abcam, ab124964, 1:2000 dilution), and cytokeratin 18 (Abcam, ab668, 1:1000 dilution). In negative controls, the primary antibody was replaced by a normal IgG (Sigma, I8765, 1:1000). A horseradish peroxidase (HRP)-conjugated secondary antibody (from MaxVision kit, kit-5005, Maxim Biotechnologies) was used for detection. Hematoxylin was used for counterstaining. The stained sections were examined under a light microscope (Leica DM2000 LED) with a digital camera.

For immunofluorescent staining or co-staining, tissue sections were prepared, as described above. In addition to the primary antibodies listed above, Alexa-488 (green) or 594 (red) labeled secondary antibodies (Invitrogen, A21202 and A11012) were used. The slides were mounted with a DAPI (4-6-diamidino-2-phenylindole) solution (SouthernBiotech, 0100-20) to stain cell nuclei and examined under a confocal microscope (Olympus, FV1000).

RT-PCR

RT-PCR experiments were done to analyze *Corin* mRNA expression in tissues, including hearts, livers and paw skins, in WT, corin KO, and corin hcKO mice. Total RNAs were isolated from the tissues using TRIzol reagents (Invitrogen, 15596018) to make first-strand cDNAs with the High Capacity cDNA Synthesis kit (Thermo Fisher, 4368814). PCR amplification of *Corin* transcripts was done using forward (5’-ATC ACT CAC AGC CAG TGT CAA-3’) and reverse (5’-TCC ACA GAG TGA TTG CTT TCC AT-3’) primers in 32 cycles of 1 min denaturation at 95°C, 1 min annealing at 55°C, and 30 s elongation at 72°C. As controls, primers for *glyceraldehyde 3-phosphate dehydrogenase* (*Gapdh*) were included in parallel reactions. Amplified PCR products were examined by agarose gel electrophoresis. Quantitative (q) RT-PCR studies were also done in footpads in WT and corin KO mice to examine levels of *Scnn1b*, encoding β-ENaC, *Cftr*, *Npr1* and *Pcsk6* mRNA expression. Sequences of the primers used are listed in Table A in S1 Text.

**Western blotting**

To examine corin protein expression in mouse paw skin, footpads, hearts (positive control), and livers (negative control) from WT and corin KO mice were homogenized in a lysis solution with 1% Triton X-100 (v/v), 50 mM Tris-HCl, pH 8.0, 150 mM NaCl, and a protease inhibitor mixture (Roche Applied Science, 4693116001, 1:100 dilution). Protein concentrations in lysates were quantified using a BCA protein assay (Thermo Scientific, 23227). Proteins were separated by SDS-PAGE and transferred to polyvinylidene difluoride membranes**.** The anti-corin antibody (1:1000 dilution) or anti-β-ENaC antibody (1:2000 dilution), described above, was used to incubate with the western blot membrane at room temperature for 2 h. After washing, an HRP-conjugated secondary antibody (Bioworld Technology, bs13278, 1:10000) was used for detection. After washing, western blots were developed with a chemiluminescent machine (Amersham Imager 600). In these experiments, Gapdh protein was analyzed to assess protein loading using an antibody from Multi Science Biotech (ab011-100, 1:5000). Antibodies used in this study are listed in Table B in S1 Text.

Sweat response

An iodine-starch painting method [5, 6] was used to examine sweat response in mice. Mice (equal numbers of males and females, 8-10 weeks old) were anesthetized with ketamine (Toronto Research Chemicals, K165300, 40 mg/kg of body weight, i.p.) and xylazine (APExBIO, B3344, 2.5 mg/kg of body weight, i.p.). A hind paw was cleaned with distilled water, dried with Kimwipe paper (Kimberly-Clark), and painted with an iodine solution (Fluka, 319007) followed by a coat of starch (Adamas, 89181a) in mineral oil (Sangon Biotech, b500301) (5 g starch/10 mL of mineral oil). Pilocarpine (AbMole BioScience, M2944, 5 mg/kg of body weight, s.c.) was injected into the paw. At 0, 1 and 2 min, photos were taken. Black dot counts (for eccrine sweat gland numbers at 1 min) and black-staining areas (for sweat excretion at 2 min) were quantified using Image-Pro-Plus 6.0 software (Media Cybernetics).

To measure sweat volume, a method with a stereomicroscope (Olympus, SZX16) with digital camera was used based on a previous publication [7]. A hind paw of anesthetized mice was rinsed with distilled water, dried with Kimwipe paper, and injected with pilocarpine, as described above. The paw was immersed in water-saturated mineral oil in a clean dish. After 10 min, photos were taken and the number and diameter of sweat droplets on footpads were quantified by Image-Pro-Plus software to calculate sweat volume.

Analysis of sweat Na^+^ and Cl^-^

Sweat samples were collected from WT and corin KO mice to analyze Na^+^ and Cl^-^ levels. Mice were anesthetized, as described above. Paws were rinsed with distilled water, dried, and injected with pilocarpine, as described above. Paws were immersed in water-saturated mineral oil in a clean dish. After 10 min, sweat droplets on footpads were mixed with double distilled water (30 μL/per 8 paws) and collected. Na^+^ and Cl^-^ concentrations were measured by an electrolyte analyzer (Shelf Scientific, 6230M) with micro Na^+^ and Cl^-^ electrodes (Shelf Scientific, ISM-146Na and LIS-146CLCM). Result from each 30-μL diluted sweat collection was recorded as one data point.

**Treatments of ENaC and CFTR inhibitors and aldosterone**

WT and corin KO mice (equal numbers of males and females, 8-10 weeks old) were fed 0.3 or 4% NaCl diet. After 2 weeks, the mice were treated with the ENaC inhibitor amiloride (Abcam, ab120281, 3 mg/kg of body weight, i.p.; daily for 5 days) [8], the CFTR inhibitor CFTR(inh)-172 (MedChemExpress, HY-16671, 0.3 mg/kg of body weight, i.p.; daily for 3 days) [9], or aldosterone (APExBIO, C4453, 0.2 mg/kg of body weight, i.p.; daily for 3 days) [10]. After the treatment, the mice were examined for their sweat responses in the iodine-starch test, as described above. Levels of sweat Na^+^ and Cl^-^ from these mice were analyzed, as described above.

**Blood pressure measurements**

A computerized non-invasive photoelectric tail-cuff system (Visitech Systems, BP-2000) was used to measure blood pressure in mice on 0.3 and 4% NaCl diets [11]. Before formal measurements, mice were trained to acclimate to the instrument and test room surroundings. The specimen platform was pre-warmed and mice were put on the platform under a cover with their tails inserted in the cuff for practice measurements, which included 5 pre-conditioning cycles and 20 regular cycles with 5 seconds between two cycles and maximal cuff pressure of 150 mmHg. After three days of practice, mean blood pressure was measured in mice with or without aimiloride (3 mg/kg of body weight, i.p.; daily for 5 day) or amlodipine (AbMole, M5139, 1.5 mg/kg; daily for 5 days) [8] treatment.

Serum aldosterone, Na^+^ and Cl^-^ measurements

Blood samples were collected from WT, corin KO, and corin hcKO mice fed 0.3 or 4% NaCl diet. Serum aldosterone levels were measured by an ELISA kit (Elabscience, E-EL-0070c), according to manufacturer’s protocols. Serum Na^+^ and Cl^-^ levels were measured by a biochemical analyzer (Roche, COBAS C 702).

**References**

1. Schwenk F, Baron U, Rajewsky K. A cre-transgenic mouse strain for the ubiquitous deletion of loxP-flanked gene segments including deletion in germ cells. Nucleic Acids Res. 1995;23(24):5080-1. Epub 1995/12/25. doi: 10.1093/nar/23.24.5080. PubMed PMID: 8559668; PubMed Central PMCID: PMCPMC307516.

2. Sohal DS, Nghiem M, Crackower MA, Witt SA, Kimball TR, Tymitz KM, et al. Temporally regulated and tissue-specific gene manipulations in the adult and embryonic heart using a tamoxifen-inducible Cre protein. Circ Res. 2001;89(1):20-5. Epub 2001/07/07. PubMed PMID: 11440973.

3. Dong L, Wang H, Dong N, Zhang C, Xue B, Wu Q. Localization of corin and atrial natriuretic peptide expression in human renal segments. Clin Sci (Lond). 2016;130(18):1655-64. Epub 2016/06/28. doi: 10.1042/cs20160398. PubMed PMID: 27343265; PubMed Central PMCID: PMCPMC5237585.

4. Cui Y, Wang W, Dong N, Lou J, Srinivasan DK, Cheng W, et al. Role of corin in trophoblast invasion and uterine spiral artery remodelling in pregnancy. Nature. 2012;484(7393):246-50. PubMed PMID: 22437503.

5. Klar J, Hisatsune C, Baig SM, Tariq M, Johansson AC, Rasool M, et al. Abolished InsP3R2 function inhibits sweat secretion in both humans and mice. J Clin Invest. 2014;124(11):4773-80. Epub 2014/10/21. doi: 10.1172/jci70720. PubMed PMID: 25329695; PubMed Central PMCID: PMCPMC4347256.

6. Tafari AT, Thomas SA, Palmiter RD. Norepinephrine facilitates the development of the murine sweat response but is not essential. J Neurosci. 1997;17(11):4275-81. Epub 1997/06/01. PubMed PMID: 9151744.

7. Song Y, Sonawane N, Verkman AS. Localization of aquaporin-5 in sweat glands and functional analysis using knockout mice. J Physiol. 2002;541(Pt 2):561-8. Epub 2002/06/04. PubMed PMID: 12042359; PubMed Central PMCID: PMCPMC2290337.

8. Wang W, Shen J, Cui Y, Jiang J, Chen S, Peng J, et al. Impaired sodium excretion and salt-sensitive hypertension in corin-deficient mice. Kidney Int. 2012;82(1):26-33. PubMed PMID: 22418978.

9. Ma T, Thiagarajah JR, Yang H, Sonawane ND, Folli C, Galietta LJ, et al. Thiazolidinone CFTR inhibitor identified by high-throughput screening blocks cholera toxin-induced intestinal fluid secretion. J Clin Invest. 2002;110(11):1651-8. Epub 2002/12/05. doi: 10.1172/jci16112. PubMed PMID: 12464670; PubMed Central PMCID: PMCPMC151633.

10. Winter C, Schulz N, Giebisch G, Geibel JP, Wagner CA. Nongenomic stimulation of vacuolar H+-ATPases in intercalated renal tubule cells by aldosterone. Proc Natl Acad Sci U S A. 2004;101(8):2636-41. Epub 2004/02/26. doi: 10.1073/pnas.0307321101. PubMed PMID: 14983061; PubMed Central PMCID: PMCPMC357002.

11. Wang C, Wang Z, He M, Zhou T, Niu Y, Sun S, et al. Krüppel-like factor 17 upregulates uterine corin expression and promotes spiral artery remodeling in pregnancy. Proc Natl Acad Sci U S A. 2020;117(32):19425-34. Epub 2020/07/29. doi: 10.1073/pnas.2003913117. PubMed PMID: 32719113.

**Table A.** **Primers used in PCR, RT-PCR and qRT-PCR**

| Gene | Locus | Primer | Sequence | Size (bp) |
| --- | --- | --- | --- | --- |
| *Cftr* | NM_021050.2 | forward | TCAGGACACTGCTTCTTCACC | 117 |
|  |  | reverse | GAACGCGGCTTGACAACTT |  |
| *Corin* | NM_016869.3 | forward | ATCACTCACAGCCAGTGTCAA | 390 |
|  |  | reverse | TCCACAGAGTGATTGCTTTCCAT |  |
| *Corin* | NM_016869.3 | forward | TGCAGATCCGAGCACCAGCGTA | *Cor*^+/+^ 2789  *Cor^flox^* 2966  *Cor^del4^* 1488 |
|  |  | reverse | AAACAGAATGAGAATCGAGC |  |
| *Corin* | NM_016869.3 | forward | TGCAGATCCGAGCACCAGCGTA | *Cor^flox^* 1746 |
|  |  | reverse | AAGGGTTATTGAATATGATCGGA |  |
| *Gapdh* | NM_001289726.1 | forward | TGTTCCTACCCCCAATGTGT | 138 |
|  |  | reverse | GGTCCTCAGTGTAGCCCAAG |  |
| *Scnn1b* | NM_001272023.1 | forward | GCCCCTGATCGCATAATCCT | 158 |
|  |  | reverse | AATTCAGTCCCAGGGTTGGC |  |
| *Pcsk6* | NM_001291184.1 | forward | GCTCCATTTTTGTTTGGGCCT | 110 |
|  |  | reverse | CTCAGTGGTGCTGCTTACGG |  |
| *Npr1* | NM_008727.5 | forward | AGACGATGGGCAGGATAGGA | 105 |
|  |  | reverse | AGCTGCTTCAGGAATTCCAAGT |  |

**Table B. Antibodies used in this study**

| Antibody | Species | Sources or ref. | Identifiers | Concentrations |
| --- | --- | --- | --- | --- |
| Anti-corin | Rabbit | Dong et al. 2016 |  | 1:500 (IHC, IF) |
|  |  |  |  | 1:1000 (Western blots) |
| Anti-corin | Mouse | Cui et al. 2012 |  | 1:500 |
| Anti-pro-ANP/ANP | Rabbit | Merck Millipore | AB2232 | 1:100 |
| Anti-NPR-A | Rabbit | Abcam | Ab14356 | 1:1000 |
| Anti-β-ENaC | Rabbit | StressMarq Biosciences | Spc-404 | 1:100 (IHC, IF)  1:1000 (WB) |
| Anti-CFTR | Mouse | Abcam | Ab2784 | 1:200 |
| Anti-pan-cytokeratin | Mouse | MXB Biotechnologies | Kit-0009 | 1:1 |
| Anti-SMA | Rabbit | Abcam | Ab124964 | 1:2000 |
| Anti-cytokeratin 18 | Mouse | Abcam | Ab668 | 1:1000 |
| Anti-IgG | Mouse | Sigma | I8765 | 1:1000 |
| Anti-IgG (mouse)-Alexa-488 | Donkey | Invitrogen | A21202 | 1:500 |
| Anti-IgG (rabbit)-Alexa-594 | Goat | Invitrogen | A11012 | 1:500 |
| Anti-IgG (rabbit) (H+L)-HRP | Goat | Bioworld Technology, | Bs13278 | 1:10000 |
| Anti-Gapdh-HRP | Mouse | Multi Science Biotech | Ab011-100 | 1:5000 |
