# Supplementary figures and images for: The protease corin regulates electrolyte homeostasis in eccrine sweat glands
Source: PLoS Biol. 2021 Feb 16;19(2):e3001090. doi: 10.1371/journal.pbio.3001090 (PMC7909636; doi:10.1371/journal.pbio.3001090)

Fig. 1F

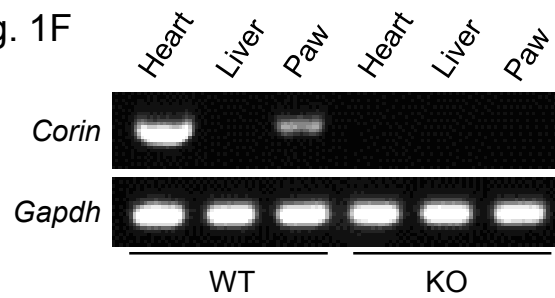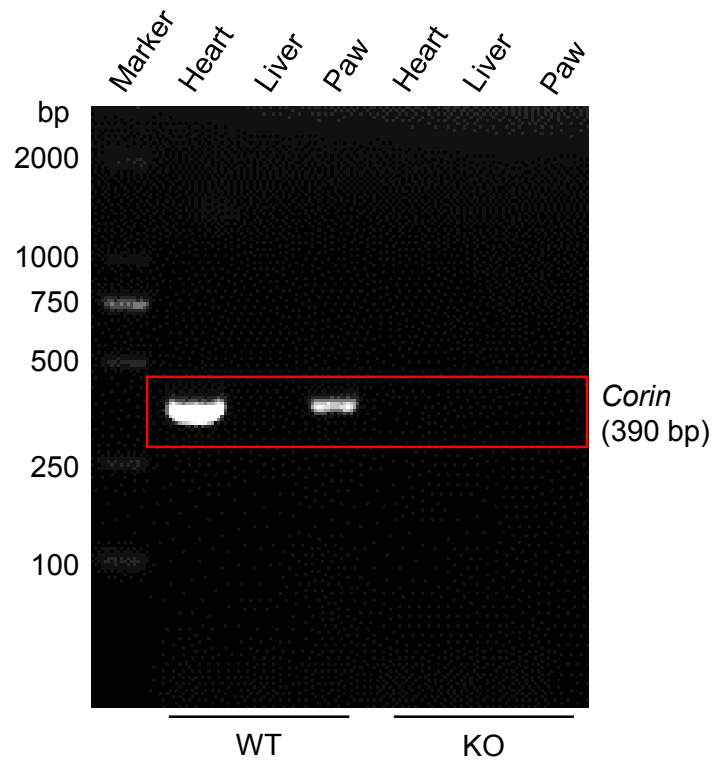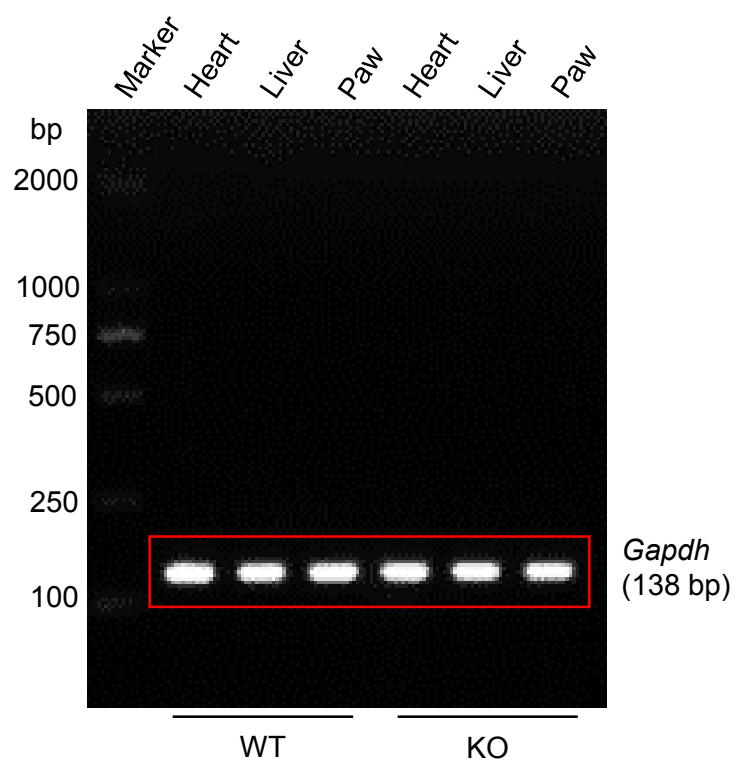

Fig. 1G

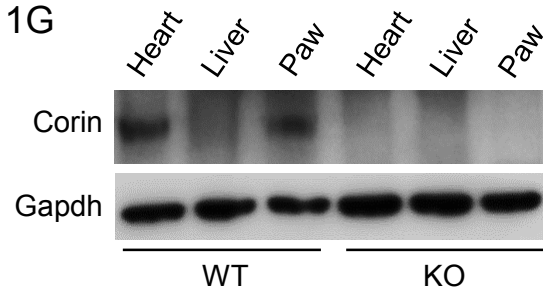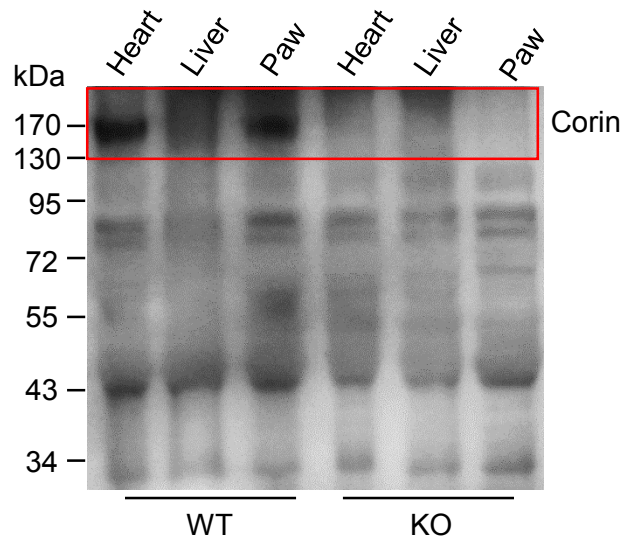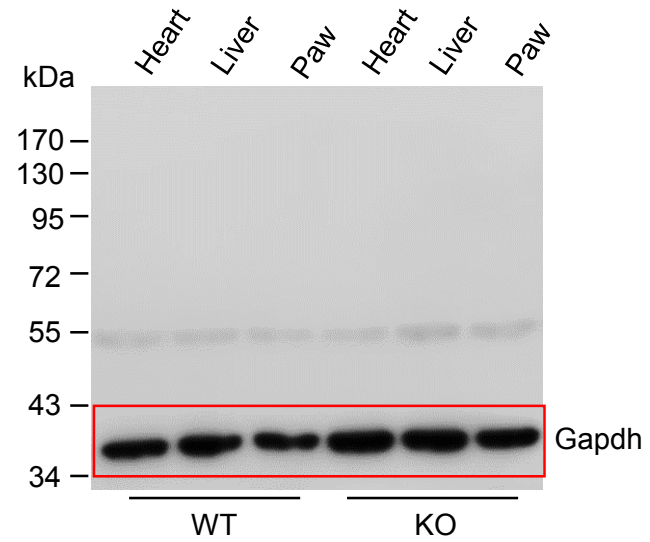

Fig. 2E

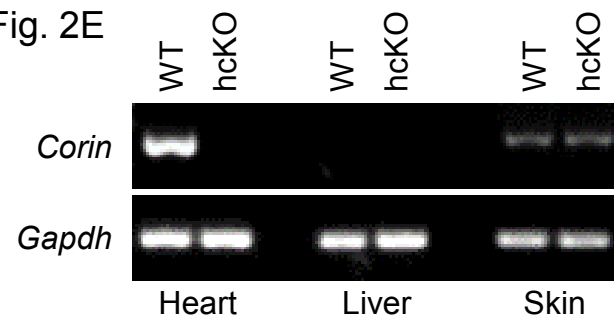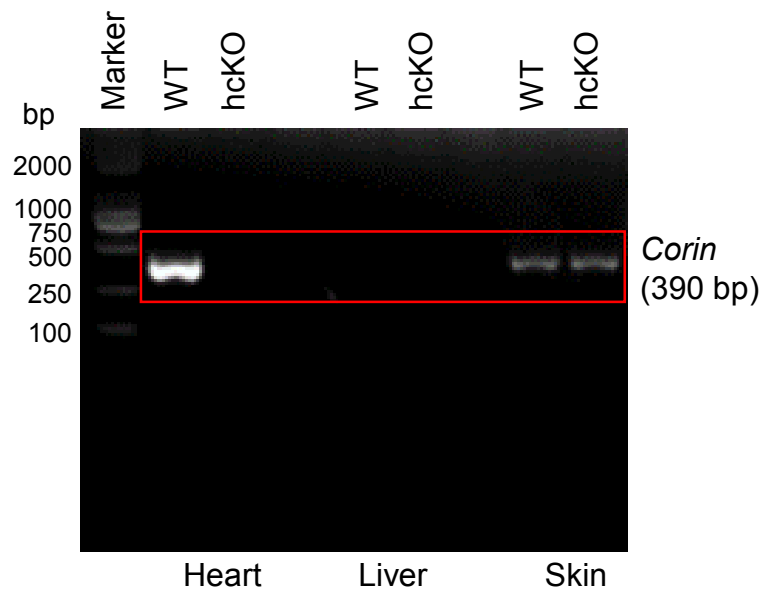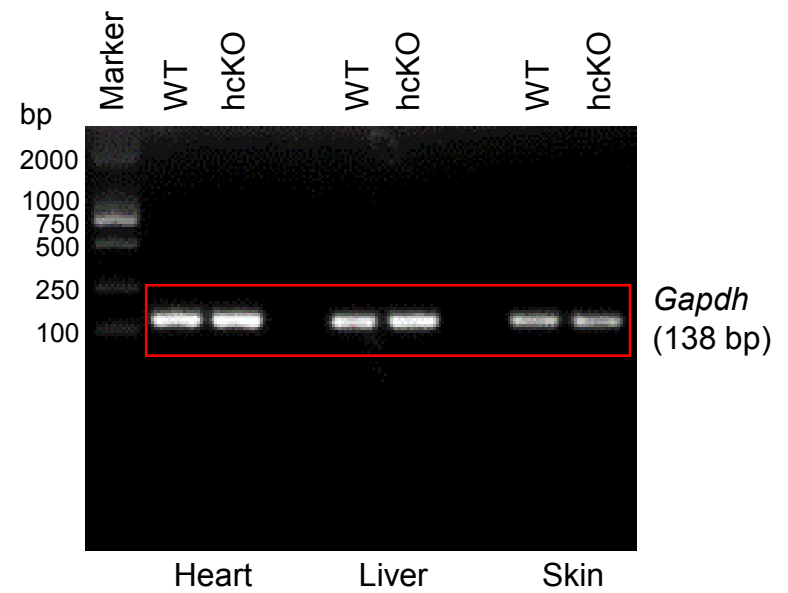

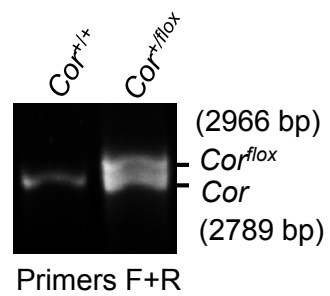

Fig. S2B

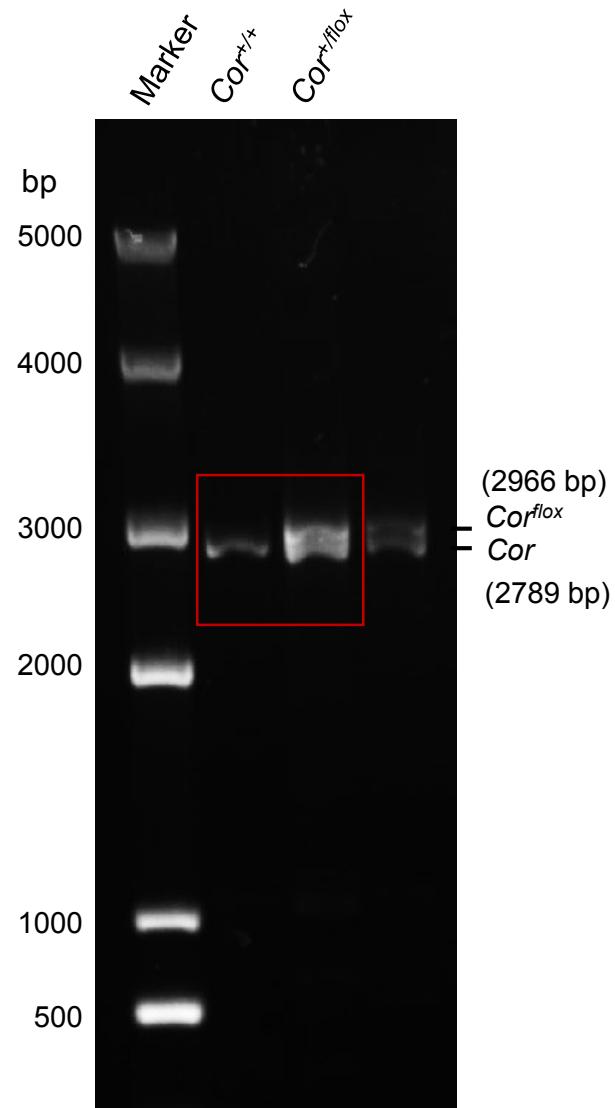

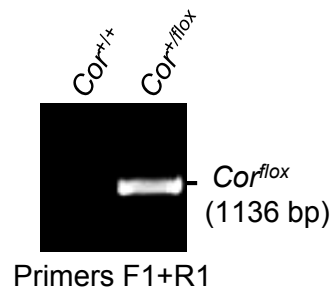

Fig. S2C

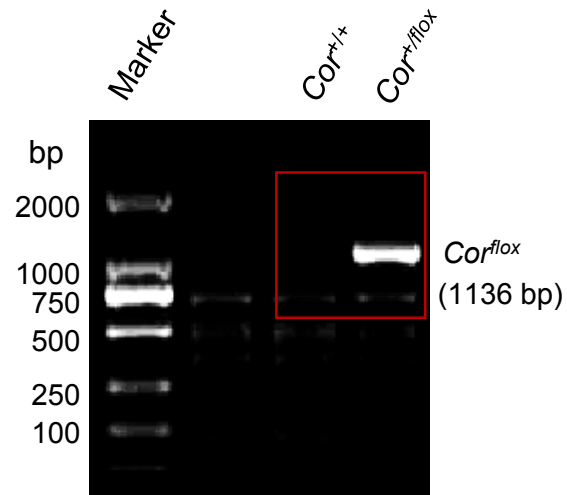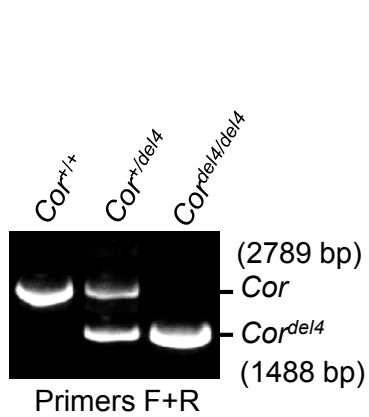

Fig. S2D

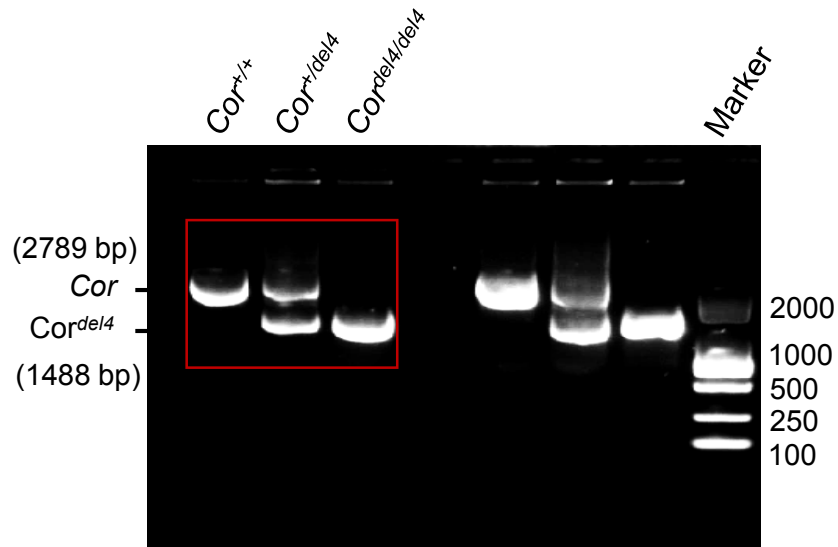

Fig. S4B

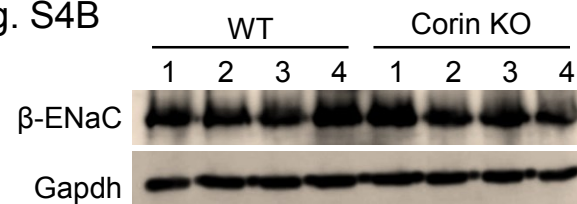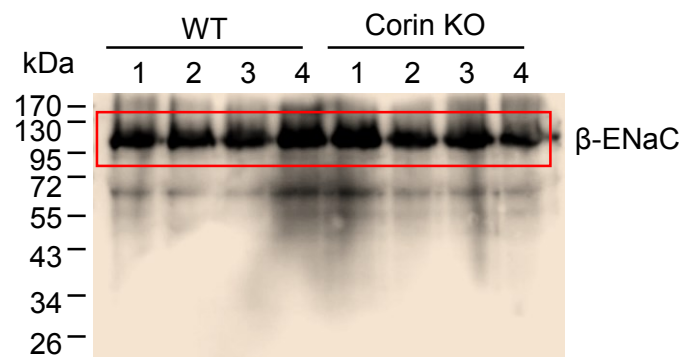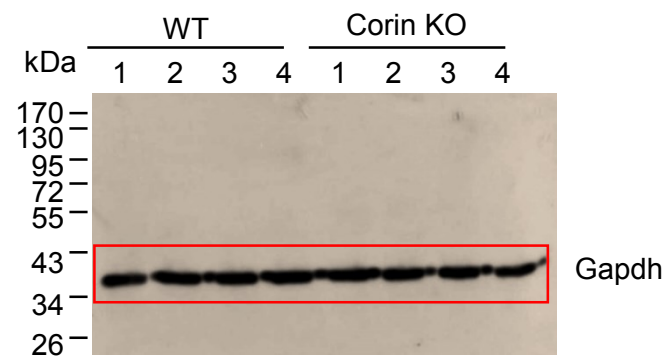

Supplement: S1 Original Blots and Gels — Raw images used for panels in Figs 1 and 2 and S2 and S4 Figs. (PDF) [file pbio.3001090.s007.pdf]
