# Supplementary material for: The protease corin regulates electrolyte homeostasis in eccrine sweat glands
Source: PLoS Biol. 2021 Feb 16;19(2):e3001090. doi: 10.1371/journal.pbio.3001090 (PMC7909636; doi:10.1371/journal.pbio.3001090)
Supplement: Attachment — Submitted filename: PLoSBiolRevLet.pdf [file pbio.3001090.s008.pdf]

November 10, 2020

Qingyu Wu, M.D., Ph.D.
Professor of Molecular Medicine
Cardiovascular & Metabolic Sciences
9500 Euclid Avenue
Cleveland, OH 44195
Office: 216-444-4351
E-mail: wuq@ccf.org

Ines Alvarez-Garcia, PhD
Senior Editor
PLoS Biology

Dear Dr. Alvarez-Garcia,

I am sending a revised manuscript, entitled "The protease corin regulates electrolyte homeostasis in eccrine sweat glands" (PBIOLOGY-D-20-02208R1), and would like to thank you and the reviewers' for the positive feedback and insightful suggestions.

To address the reviewers' concerns, we have done additional experiments that were suggested by the reviewers. Our responses to the reviewers' specific concerns are described below.

Reviewer #1:
Essential revisions requested:
No morphological differences were found between footpads of corin KO mice and controls. The authors should also comment on whether or not histological differences were observed in eccrine sweat gland structure and numbers.

Response: We thank the reviewer for the suggestion and have revised the sentence to indicate that we did not find morphological differences regarding eccrine sweat gland structure and numbers between WT and corin KO mice (page 5, second paragraph, lines 1-3).

More information should be provided on the heart-conditional corin KO. How was excision efficiency addressed? Did this mouse display hypertension as observed in the global KO? What is known about plasma electrolytes in global and cardiac specific corin KO mice?

Response: As shown in Fig. 2E, RT-PCR detected *Corin* mRNA in the kidney but not the heart in heart-conditional corin KO (hcKO) mice, indicating that the *Corin* gene was selectively disrupted in the heart.

Like corin global KO mice, corin hcKO mice are hypertensive (systolic blood pressure: 117.8 ± 1.5 mmHg *vs*. 107.0 ± 1.7 mmHg in WT mice, $p<0.001$, n = 8 per group; or 117.4 ± 2.9 mmHg in corin global KO mice, $p=0.934$, n = 8 per group). Plasma Na^+ and Cl^- levels were not statistically different among WT, corin KO, and corin hcKO mice. We include this information in Supporting Information (Supplementary Materials and Methods, page 1, second paragraph, lines 4-10).

Were the experiments littermate controlled? If not, how were wild-type controls generated, where were they bred, and what was their relation to the knockout mice?

Response: WT and KO mice were not littermates, but were bred and housed in the same room at a specific-pathogen-free facility. Age- and gender-matched mice were used in each experiment. We have revised the Supporting Information to include this information (Supplementary Materials and Methods, page 2, first paragraph, lines 3-6).

Statistical analysis: Were normality tests performed before t-tests? One-way ANOVA is used for some experiments where the effect of both treatment and genotype are assessed. Does each symbol in bar graphs represent a different mouse or were several paws from the same mouse studied or the same mouse studied more than once?

Response: Normality tests were performed before *t* tests. We have revised the Statistical Analysis section to include this information (page 13, third paragraph, lines 1-4). Each symbol in bar graphs represents the data from one mouse except the bar graphs for sweat Na^+ and Cl^-, in which each symbol represents the data from eight paws from two mice. We have revised the figure legends to make this clear.

Non-essential revisions suggested:
As pointed out by the authors, sweating is important to prevent overheating. Have they addressed if corin KO mice are impaired in their ability to maintain core body temperature with increasing ambient temperature? Although not essential, such data would corroborate the model.

Response: Unlike in humans, paw sweat glands in mice are less important in regulating core body temperature. We did not measure the core body temperature with increasing ambient temperature in our study.

Reviewer #2:
The introduction does not provide the essential information for understanding the work presented. Please expand on corin, ANP and its receptors and what is known about ANP regulating ENaC and the function of ENaC (e.g. in kidney).

Response: We have revised the Introduction to include the information of corin, ANP and NPR-A in regulating ENaC in the kidney (page 3, third paragraph, lines 1-4).

Page 4: explain why keratin was used (epithelial marker?). Page 4: "By (not "in") immunohistochemistry". Page 4: Fig.1J: The NPR-A signal seems much less in the corin KO

compared to wt. May be I missed it, but were NPR-A expression levels determined for wt vs ko? This seems important, since NPR-A activity is linked to ENaC activity and the authors need to assure that the corin ko mice have equal levels of NPR-A as the wt.

Response: Keratin is an epithelial marker. We have revised the Figure 1 legend to clarify this (page 19, figure legend). “In immunohistochemistry” has been changed to “By immunohistochemistry”, as suggested (page 4, last paragraph, line 5).

Immunostaining signals are not best for quantitative comparisons. We performed additional quantitative RT-PCR, which showed similar levels of *Npr1* mRNA in paw skins between WT and corin KO mice. The new data is shown in new Fig. S3C.

Page 5: please explain the physiologic meaning of the "black dots" and how pilocarpine functions. Very few readers will be familiar with sweat gland physiology and this particular experimenatl system, which is heavily used in the paper. Also, it appears that the observed corin KO phenotype (reduced sweat excretion, Na and Cl) is entirely dependent on the treatment with pilocarpine and is not observed without it. Therefore, a clear explanation on pilocarpine function and mechanism should be provided. The fact that in the absence of pilocarpine no phenotype was observed raises questions about the physiological relevance of the pilocarpine-dependent reductions; therefore, the authors need to explain and comment on this.

Response: The iodine-starch test to examine sweat response in mice is used by many investigators, as cited in our manuscript (refs. #22 and 23). We have revised the text to provide more information about pilocarpine and black dots in the Results (page 5, second paragraph, lines 4-5) and the Materials and Methods (page 12, second paragraph, lines 2-4).

Page Fig.2: The legend should indicate that pilocarpine was used for Fig. 2I and J.
Fig. 2H shows a significant (p 0.009) reduction with normal diet, but the authors only mention reduction with the high salt diet. Why? Also, the authors should indicate the % reductions here and in other figures as well.

Response: The legend for Fig. 2I and J has been revised to indicate pilocarpine injection. The text has been revised to indicate that the reduction was observed in corin KO mice on both normal- and high-salt diets. The percentages of the reduction are also described (page 6, first paragraph, last 4 lines).

Page 6: The authors show that ENaC expression is similar in wt and ko mice, but that ENaC activity is increased in ko. I thought that corin/ANP regulate the transcription of ENaC and that corin-deficiency increases ENaC transcripts (Zhou, Wu Current Hypertension Rep 2012; Theilig,

Wu Am.J.Physiol.Renal Physiol. 2015). If true, then the results need some explanation. If the authors conclude that there is another mechanism by which corin/ANP regulates ENaC, then this needs to be explained and discussed.

Response: Previously, increased ENaC expression was reported in the kidney of corin KO mice (Polzin D. et al Kidney Int 2010). In this study, however, we found that ENaC mRNA levels (*Scnn1b*) in paw skins between WT and corin KO mice were similar, as indicated by quantitative RT-PCR (Fig. S4A). We performed additional western blotting, which also showed similar β-ENaC protein levels in paw skins between WT and corin KO mice. The new western blotting data is included in new Fig. S4B.

Page 6, Fig. S4B. the effect of amiloride seems minimal compared to amlodipine. Please comment.

Response: Indeed, the reduction on blood pressure in corin KO mice treated with amiloride was less compared with that in mice treated with amlodipine, a calcium channel blocker. Because we did not compare the potency and dose-response curves of amiloride and amlodipine in mice, it is difficult to directly compare the effects of these two drugs on mouse blood pressure.

Page 7, fig. 3C,D: what are the Na and Cl levels w/o aldosterone?

Response: Sweat Na^+ and Cl^- levels without aldosterone are presented in Fig. 2I and J, showing reduced sweat Na^+ and Cl^- levels in corin KO mice. After aldosterone treatment, the difference between WT and corin KO disappeared, indicating a role of corin in antagonizing aldosterone function in eccrine sweat glands.

Page 7: To open the discussion with a topic that was not experimentally pursued seems a bit odd.

Response: In the Discussion, we started with a discussion about our results in this study. It is unclear if this is what the reviewer referred to. In the second paragraph, we discussed previous findings of ANP in salt glands in other species, which, we believe, is relevant to our study and may be interesting to general audience.

Page 9: I don't quite understand the conclusion made in last sentence in 2nd paragraph. To me the conclusion would be that the presented results contradict the studies with exogenous ANP in that reduced ANP in corin ko mice has the same effect as addition of exogenous ANP (i.e. increased CFTR activity). In addition, ANP actually seems to have a role in regulating CTFR activity, since the corin ko have increased CTFR activity. Also, if the CTFR levels are the same

in wt and ko, how then does ANP regulate CTFR activity? Please clarify this and discuss it better.

Response: We appreciate the reviewer's point and have revised the text to explain our results and point out the apparent difference between our data and previously published results in different tissues from different species (page 10, first four lines).

Fig.S3. Some labeling of pictures or explanation in figure legend may be helpful for readers unfamiliar with sweat gland/skin histology, e.g. indicate individual sweat glands, point out that staining is in epithelial layer etc.

Response: We have revised the figure legend to indicate sweat glands and positive epithelial cells (Fig. S3 Legend).

Reviewer #3:
Corin is a membranal enzyme and is produced by conversion of procorin into corin by PCSK6. It is very essential to examine the status of PCSK6 in the studied models. The presence of PCSK6 may strength the authors conclusion that a whole machinery of ANP production present in the eccrine sweat glands of experimental and human origin. In addition, it is of interest how high or low salt affects not only corin expression, but also PCSK6.

Response: PCSK6 is expressed ubiquitously. As the reviewer suggested, we performed quantitative RT-PCR and found similar levels of *Pcsk6* mRNA expression in paw skins between WT and corin KO mice either on a normal-salt diet or a high-salt diet. As this study is not about corin activation, we include this data in a separate file for Reviewers-Only (Panel A in the Reviewers-Only file).

The authors examined the impact of aldosterone on sweat and salt excretion in wild-type and corin deficient mice. I wonder whether spironolactone block these effects. This protocol supposed to validate the observed inhibitory impact of aldosterone on salt and sweat excretion by eccrine sweat glands. Moreover, measurement of aldosterone levels in wild and corin KO mice on low and high salt diet should be measured.

Response: Previously, we found that serum aldosterone levels were reduced in corin KO mice, compared to those in WT mice, either on a normal-salt diet or a high-salt diet (Wang W, et al. Kidney Int 2012;82:26-33). Since the corin KO mice used in this study were made by a different strategy, we did the experiment suggested by the Reviewer. Again, we found reduced serum aldosterone levels in the corin KO used in this study. As the results are not new, we included the data in Panel B in the Reviewers-Only file). We did not perform additional experiments to test the effect of spironolactone on sweat responses in WT and corin KO mice.

It is unclear whether the "n" represents number of analyzed, fields or animals. The number of animals in each studied group should be indicated in both the text and figure legends.

Response: As indicated above in the response to Reviewer 1, the "n" refers to the number of mice, except the bar graphs for sweat Na^+ and Cl^-, in which each symbol represents the data from eight paws from two mice. We have revised the figure legends to make this clear.

I wonder, if the authors have measured Na^+ and Cl^- levels in the circulation of the studied groups of animals.

Response: Previously, we reported similar plasma Na^+ and Cl^- levels in WT and corin KO mice (Wang W, et al. Kidney Int 2012;82:26-33). As the Reviewer suggested, we measured serum Na^+ and Cl^- levels in WT, corin KO, and corin hcKO mice used in this study. We found similar levels of serum Na^+ and Cl^- among WT, corin KO, and corin hcKO. The data are included in the Supporting Information (Supplementary Materials and Methods, page 1, last three lines).

Reviewer #4:
Introduction is not informative. Rather than emphasizing Corin function in hair color, the authors should introduce Corin/ANP/ENaC function in the kidneys. Also, the similarities and dissimilarities of Corin function in kidney and sweat glands should be compared in the Discussion section.

Response: We thank the Reviewer for the suggestion and have revised the Introduction (page 3, third paragraph, lines 2-4) and the Discussion (page 8, Discussion, first paragraph, last six lines) to discuss the corin/ANP/ENaC function in the kidney.

In the present study, the authors analyzed the whole-body Corin KO mice, but not skin-specific KO mice. The whole-body KO mice have previously shown retention of sodium and water because of altered kidney function, which may affect sweat secretion directly or indirectly. The authors should provide a rationale of using whole-body KO, rather than skin-specific KO mice for their study.

Response: The reviewer's point is well taken. At this time, we do not know the tissue-specific transcriptional mechanism underlying corin expression in the eccrine sweat glands. It is unknown if eccrine sweat gland-specific Cre mice are available. We have revised the Discussion to point out that more studies in eccrine sweat gland-specific KO mice will be important to verify our results (page 8, Discussion, first paragraph, last sentence).

The authors analyzed Corin function in the sweat duct. But the Corin cascade is highly expressed in the secretory portion. The study will be more complete if the authors analyze the

biological meaning of Corin expression in the sweat secretory portion.

Response: Indeed, the significance of corin expression in the sweat secretory portion remains unknown. It is technically challenging to distinguish the role of ductal *vs.* secretory portion of corin in the eccrine sweat glands. We have revised the Discussion to point out that the significance of corin expression in the sweat secretory portion remains to be determined (page 9, second paragraph, lines 5-7).

ENaC was reported to be upregulated in Corin KO kidneys, which was responsible for sodium retention. However, ENaC expression level in Corin KO sweat glands was comparable with WT controls, as shown in Fig 3A and 3B in this manuscript. The authors should quantify the ENaC expression by WB etc to show that ENaC is indeed regulated by Corin in sweat glands.

Response: As discussed above in our responses to Reviewer 2, increased ENaC expression was reported in the kidney of corin KO mice (Polzin D. et al Kidney Int 2010). In this study, we found that ENaC mRNA levels (*Scnn1b*) in paw skins were comparable between WT and corin KO mice, as shown by quantitative RT-PCR (Fig. S4A). Following the Reviewers' suggestion, we performed additional western blotting, which also showed similar β-ENaC protein levels in paw skins between WT and corin KO mice. The new western blotting data is included in new Fig. S4B).

To understand the possible effect of circulating Corin on sweat glands, the authors analyzed heart-specific Corin KO mice. However, Corin is also highly expressed in the kidneys and other tissues. The authors should measure circulating Corin in hcKO mice by ELISA etc to avoid the possibility of "contamination" from non-heart tissues.

Response: As indicated above in our response to Reviewer 1, corin hcKO mice are hypertensive. Thus, it is unlikely that circulating corin from other non-cardiac tissues such as kidneys may substitute the function of cardiac corin. At this time, ELISA kits for mouse corin are not available, making it difficult to measure circulating corin in mice. We have revised the Supplementary Materials and Methods to include the information on blood pressure in WT, corin KO, and corin hcKO mice (Supplementary Materials and Methods, page 1, second paragraph, lines 4-7).

Figs 2-4: It will be more convincing if the authors show sweat volume in addition to sweat area.

Response: As shown in Fig. 2A-D, the data of sweat area in the iodine-starch test are consistent with the data from sweat volume measurements. Because measuring sweat volume is more time-consuming, we did not measure sweat volume in all experiments.

Spelling: Page 8, the second line from the bottom, "in" should be changed to "is". Page 5 of the supplemental information, "aimiloride" should be changed to "amiloride". Page 10 of the supplemental information, "injection" should be changed to "injected".

Response: We thank the Reviewer for pointing these out. On previous page 8, in the second line from the bottom, "in" probably is correct. All other typos are fixed.

Together with the new experimental data and the revision of the text, we believe that we have adequately addressed all of the concerns by the reviewers. We would like to thank the reviewers for helping us to significantly improve this manuscript.

Sincerely yours,

Qingyu Wu, MD, PhD
