# Supplementary material for: The protease corin regulates electrolyte homeostasis in eccrine sweat glands
Source: PLoS Biol. 2021 Feb 16;19(2):e3001090. doi: 10.1371/journal.pbio.3001090 (PMC7909636; doi:10.1371/journal.pbio.3001090)
Supplement: Attachment — Submitted filename: Responses.pdf [file pbio.3001090.s009.pdf]

Cleveland Clinic
Lerner Research Institute

December 24, 2020

Ines Alvarez-Garcia, PhD
Senior Editor
PLoS Biology

Qingyu Wu, M.D., Ph.D.
Professor of Molecular Medicine
Cardiovascular & Metabolic Sciences
9500 Euclid Avenue
Cleveland, OH 44195
Office: 216-444-4351
E-mail: wuq@ccf.org

Dear Dr. Alvarez-Garcia,

I am sending a revised manuscript, entitled "The protease corin regulates electrolyte homeostasis in eccrine sweat glands" (PBIOLOGY-D-20-02208R2), and would like to thank you and the reviewers for the possible acceptance of the manuscript.

Based on your instruction, we have made additional changes in the manuscript, as described below.

<u>ETHICS STATEMENT:</u>
<u>Please include the numbers of the animal care and human tissue license.</u>

Response: The protocol numbers are included (page 11, first and second paragraphs, respectively).

<u>Please confirm that all clinical investigation have been conducted according to the principles expressed in the Declaration of Helsinki.</u>

Response: A statement has been included to indicate that the study was conducted according to the principles expressed in the Declaration of Helsinki (page 11, first paragraph, line 3).

<u>DATA DEPOSITION:</u>
<u>Regardless of the method selected, please ensure that you provide the individual numerical values that underlie the summary data displayed in the following figure panels as they are essential for readers to assess your analysis and to reproduce it:</u>

<u>Fig. 2B, D, G-J; Fig. 3C-F, I-L; Fig. 4A-D; Fig. S3C, G and Fig. S4A-F</u>

Response: We have included in 'Supporting Information' an excel file, labeled 'S1 Data', which includes multiple sheets with the original numerical values for Fig. 2B, D, G-J; Fig. 3C-F, I-L; Fig. 4A-D; Fig. S3C, G and Fig. S4A-F. Each data sheet is labeled as Fig#_Data.xls. We have also revised the figure legends to indicate that "The original numerical values are in S1 Data".

<u>BLOT AND GEL REPORTING REQUIREMENTS:</u>

Response: In 'Supporting Information', we have included a PDF file, labeled as 'Original Blots

and Gels', which contains the original, uncropped western blots and gels for Figs. 1F, 1G, 2E, S2B-D, and S4B.

BLURB

A blurb has been included as a separate file, labeled as 'Blurb', which summaries our findings: "Sweating is a basic skin function in body temperature control. Salt excretion and reabsorption in sweat glands are essential for salt-water balance. Corin has been identified as a key enzyme in regulating salt excretion in the skin".

In additional to changes indicated above, we have made two minor changes Fig. S3:

In panel C, the original *p* value (0.582) has been changed to 0.273. The original value was calculated with One-way ANOVA, inadvertently. The new value was calculated with Student's *t* test.

In panel G, the original *p* value (0.670) has been changed to 0.649. The original value was calculated by Mann-Whitney analysis in Graphpad and the new value was calculated by Mann-Whitney in SPSS, as described in the Methods.

These corrections do not change the original conclusions described in the text.

We would like to thank you and the reviewers for helping us to significantly improve this manuscript.

Sincerely yours,

Qingyu Wu, MD, PhD
